# Non-dialytic management of acute kidney injury in newborns

**DOI:** 10.15171/jrip.2017.01

**Published:** 2016-10-29

**Authors:** Vishal Pandey, Deepak Kumar, Prashant Vijayaraghavan, Tushar Chaturvedi, Rupesh Raina

**Affiliations:** ^1^Department of Pediatrics and Neonatology, University of Kansas Hospital, Kansas City, KS, USA; ^2^Department of Pediatrics and Neonatology, MetroHealth Medical Center, Cleveland, OH, USA; ^3^Division of Nephrology, Department of Internal Medicine and Research Cleveland Clinic Akron General, Akron, OH, USA 4Akron Children’s Hospital, Cleveland, OH, USA; ^4^Akron Children’s Hospital, Cleveland, OH, USA

**Keywords:** Acute kidney injury, Non-dialytic management, Newborn, pRIFLE, Neonates

## Abstract

Treating acute kidney injury (AKI) in newborns is often challenging due to the functional immaturity of the neonatal kidney. Because of this physiological limitation, renal replacement therapy (RRT) in this particular patient population is difficult to execute and may lead to unwanted complications. Although fluid overload and electrolyte abnormalities, as seen in neonatal AKI, are indications for RRT initiation, there is limited evidence that RRT initiated in the first year of life improves long-term outcome. The underlying cause of AKI in a newborn patient should determine the treatment strategies to restore appropriate renal function. However, our understanding of this common clinical condition remains limited, as no standardized, evidence-based definition of neonatal AKI currently exists. Non-dialytic management of AKI in these patients may restore appropriate renal function to these patients without exposure to complications often encountered with RRT.

**Implication for health policy/practice/research/medical education:**

Treating acute kidney injury (AKI) in newborns is often challenging due to the functional immaturity of the neonatal kidney. Because of this physiological limitation, renal replacement therapy in this particular patient population is difficult to execute and may lead to unwanted complications.

## Introduction

Acute kidney injury (AKI) is a common occurrence in newborns admitted to Neonatal Intensive Care Units (NICUs) (1-5). Recognizing neonates who are at risk for AKI is important, not only for prevention, but also for early diagnosis and treatment. Common renal replacement modalities used in the NICU to support neonatal patients with AKI include peritoneal dialysis (PD), continuous renal replacement therapy (CRRT), and intermittent hemodialysis (IHD) (6). However, RRT is not only technically difficult, but also associated with high rates of complications (7-12). The neonatal age group presents unique challenges in the management of AKI namely due to nephron development, multifactorial causation, and limitation of treatment options due to technical difficulties arising from the size of the vessels and peritoneal space (8,13). These distinct sets of pathophysiological considerations have initiated a push toward redefining the definition of AKI for neonates, rather than extrapolating the data from adult and pediatric criteria (14). In this paper we intended to review, non-dialytic management of AKI in newborns. In this review we also address the management of fluid and electrolyte imbalance, nutritional requirements, and management of hypotension through non-dialytic means.

## Materials and Methods

For this review, we used a diversity of sources by search­ing through PubMed/Medline, Scopus, EMBASE, Web of Science, EBSCO and directory of open access journals (DOAJ). The search was conducted using combination of the following key words and or their equivalents; acute kidney injury, non-dialytic management, newborn, pRIFLE and neonates. Titles and abstracts of review articles, clinical trials, cohort studies, case-control studies, and reports that held relevance to the intended topic were studied.

## Goal of AKI management

The goal of AKI management in newborns is to maintain homeostasis until the renal functions return, and is accomplished by addressing fluid and electrolyte imbalance, nutritional needs, and acidosis (15,16). Unfortunately, available data on the long-term outcome of neonatal AKI patients is limited (15,17,18). Additionally, managing neonates with dialysis is more expensive than management in adults, and outcomes of treatment remain ambiguous due to minimal evidence-based studies (17,18). Therefore, non-dialytic management of neonatal AKI may be a more suitable alternative to RRT in this particular patient population.

## Incidence

Studies have shown that pre-term infants (very low birth weight and extremely low birth weight) (5,19,20), sick near-term/term asphyxiated infants (21,22), those who received extracorporeal membrane oxygenation (ECMO), infants with neonatal sepsis (18), and newborns with congenital heart disease requiring surgery (23,24) experience high rates of AKI as seen in Table 1. Additionally, the risk of mortality and/or developing chronic kidney disease (CKD), following an episode of AKI, is increased in these patients (16). However, due to heterogeneity in study design (i.e. sample size, sample population, use of control population, etc.) and AKI definition used, it is difficult to determine epidemiological relationships between rate of AKI incidence and long-term outcome in neonates (25,26).

**Table 1:** AKI Incidence in various neonatal populations and the AKI definition used for diagnosis

**Population**	**AKI incidence**	**AKI definition**
Pre-term VLBW	18%	Modified KDIGO
Pre-term ELBW	12.5%	Modified KDIGO
Sick near-term/term	18%	Modified KDIGO
Asphyxiated newborn	38%	Modified KDIGO
ECMO	71%	RIFLE criteria
Sepsis	26%	
Cardiac surgery	62%	AKIN criteria

Source: Reference 16.

## Diagnosis

### Surrogates to glomerular filtration rate; serum creatinine

In newborns, AKI is difficult to define due to persistence of maternal creatinine during the first 3 days of life and overall low glomerular filtration rate (GFR) and maturational differences in the creatinine absorption at the level of the proximal tubules (2,3,14,16,27-29). In the past an absolute value of serum creatinine >1.5 mg/dL was considered to represent acute renal failure (ARF). AKI is now defined as abrupt decrease in the kidney function that includes, but is not limited to ARF (30). Serum creatinine is a reflection of kidney functions and does not accurately represent kidney injury. Moreover, a rise in the creatinine may not occur immediately after injury, but when 25% to 50% of the kidney functions are lost (31). The KDIGO concept of AKI incorporates urine output and duration in addition to changes in serum creatinine. This can be seen in Table 2. Severity of AKI is then defined by taking all three factors into account. Per consensus definition, an increase in serum creatinine of 0.3 mg/dL above the baseline within 48 hours or 1.5 to 1.9 times the baseline serum creatinine value is considered the earliest stage of AKI (3,14,16,27,29,30). Inclusion of such sensitive criteria into the definition are intended for early detection of kidney injury, which serves to potentially mitigate ongoing damage due to reversible causes. Based on a study conducted by Askenazi et al, due to the lower baseline serum creatinine in neonates, an increase of this magnitude is often more detrimental in neonatal patients than in pediatric patients (32). Following this study, Jetton and Askenazi proposed modification of KDIGO criteria that could be used for neonatal patients (Table 3) (19,33). AKI can be oliguric or non-oliguric; multiple reports have demonstrated that the presence of oliguria and accompanying fluid overload are extremely poor prognostic factors in children with AKI (1,21,27,34). The modified KDIGO criteria takes into consideration that neonates often have non-oliguric renal failure, which makes urine output a less sensitive indicator of AKI (33). Overall prognosis is determined by the severity of AKI, which has been categorized into progressively worsening stages, taking serum creatinine, urine output and the duration of oliguria into account (30).

**Table 2:** KDIGO criteria for AKI

**AKI stage**	**Serum creatinine (SCr)**	**Urine output**
1	1.5-1.9 times baseline OR > 0.3 mg/dL increase	< 0.5 mL/kg/h for 6-12 hours
2	2.0–2.9 times baseline	< 0.5 mL/kg/h for > 12 hours
3	3.0 Times baseline OR Increase in SCr to > 4.0 mg/dL OR Initiation of RRT OR Decrease in eGFR to < 35 mL/min/1.73 m^2^ in patients < 18 years	< 0.3 mL/kg/h for >24 hours OR Anuria for > 12 hours

**Table 3:** Modified KDIGO for use in neonatal patients

**AKI Stage**	**Serum Creatinine (SCr)**
0	No change or rise <0.3 mg/dL
1	Increase SCr 0.3 mg/dL OR Increase SCr 150%-200% from previous trough value
2	Increase SCr 200%-300% from previous trough value
3	Increase SCr 300% from previous trough value OR 2.5 mg/dL OR Initiation of RRT

## Oliguria

Oliguria in newborns has traditionally been defined as urine output <0.5 to 1 mL/kg/h (26,29,35-40). Duration of oliguria is also taken into consideration, and the combined score is used to classify AKI into an increasing degree of renal impairment per the pRIFLE scoring (Table 4). In a prospective single-center cohort study by Bresolin et al, pRIFLE was tested in an ICU setting for AKI prevention (41). Within the younger patient cohort in this study, use of pRIFLE resulted in 72.4% of these patients being diagnosed with AKI on day one of ICU admission (41). This study, along with others (42-44), has validated the sensitivity of pRIFLE diagnostic criteria for AKI encountered in the ICU. Although this is an extrapolation of the adult scoring system, various reports suggest that higher pRIFLE scores are associated with poor prognosis (45). Fluid overload associated with oliguria seems to be an independent risk factor for poor prognosis (21).

**Table 4:** RIFLE, pRIFLE, and nRIFLE criteria for AKI

	**Serum Creatinine** ** (SCr)**	**Urinary Output and Duration**		
		**RIFLE**	**pRIFLE**	**nRIFLE**
Risk (R)	SCr X 1.5	< 0.5 mL/kg/h (6 h)	< 0.5 mL/kg/h for (8 h)	< 1.5 mL/kg/h (24 h)
Injury (I)	SCr X 2.0	< 0.5 mL/kg/h (12 h)	< 0.5 mL/kg/h for (16 h)	< 1.0 mL/kg/h (24 h)
Failure (F)	SCr X 3.0 or > 4 or Acute rise > 0.5 mg/dL	< 0.3 mL/kg/h (24 h) OR Anuric (12 h)	< 0.3 mL/kg/h (24 h) OR Anuric (12 h)	< 0.7 mL/kg/h (24 h) OR Anuric (12 h)
Limitation (L)	Loss of kidney function for 4 weeks			
End stage (E)	Loss of kidney function > 3 months			

Source: Reference 14.

However, pRIFLE does not take into consideration neonatal specific characteristics, which are more pronounced in preterm patients (15). Because these patients are so fragile, few serum creatinine measurements are taken, making baseline values difficult to establish (14). Determining actual urinary output is also complicated as urinary catheters are rarely placed in newborns (14). In a study conducted by Bezerra et al, pRIFLE criteria was used to evaluate patients in the NICU (26). In this study, worsening oliguria with urine output less <1.5 mLkg/h was associated with increasing mortality in critically ill newborn infants in the NICU. They recommended adopting higher urine output (<1.5 mL/kg/h) values for the newborn than those in the pRIFLE (<0.5 mL/kg/h) (26). Increased urinary output may be explained by greater total body water in newborns compared to other patient populations (26). Based on findings in this study, nRIFLE was established with urinary output values that accommodate findings in neonatal AKI patients (14). Table 4 compares RIFLE, pRIFLE, and nRIFLE criteria for urinary output and duration of oliguria.

The cause of oliguria warrants investigation since oliguria can be present without AKI, especially in preterm infants. Hypovolemia, with or without hypotension, results in increased vasopressin secretion leading to free water reabsorption through the aquaporin channels and low volume concentrated urine (10,16). Similarly, syndrome of inappropriate secretion of antidiuretic hormone (SIADH) may result in decreased urinary output with resultant dilutional hyponatremia. Urine osmolarity and measurement of fractional excretion of sodium (FENA) are useful indices to determine the fluid status as well as the renal concentrating ability (46). The results of FENA should be interpreted with caution in preterm infants with immature kidneys since the kidneys do not have the mature distal tubular functions (46). In order to rule out obstructive causes of oliguria/anuria in this age group, kidney, bladder and ureter ultrasounds are recommended to rule out posterior urethral valves, urethral agenesis, urethrocele and functional bladder outlet obstruction due to commonly used medications like morphine (47). Renal scans with Doppler are useful in the diagnosis of conditions like renal vein thrombosis, and very rare conditions like renal arterial stenosis/aortic thrombus (47). Furthermore, resistive indices in the kidneys are indicative of established AKI.

## Identifying etiology and other contributing factors

In the majority of newborns with AKI, multiple nephrotoxic factors are present and have a cumulative effect leading to AKI (48). Although seen in preterm and term neonates, the etiology of AKI differs between these two groups. In term neonates, leading causes of AKI are congenital anomalies of the kidneys and urinary tract (CAKUT), post-surgical complications, hypothermia, obstructive uropathies and systemic (birth asphyxia)/metabolic derangements (1-3,7,27,34,35,49,50). While in the preterm population, poor renal perfusion due to hypotension, sepsis, necrotizing enterocolitis (NEC), patent ductus arteriosus (PDA) and nephrotoxic medications play a major role (5,26,27,36,51,52). In addition, neonates have persistence of maternal creatinine (53) and massive fluid shifts associated with post-natal weight loss.

## Nephrotoxic medications

Table 5 lists the common nephrotoxic medications used in the neonatal population (16). Levels of some of the medications are monitored routinely in the NICU, but if the levels cannot be measured for dose adjustment, those medications should be avoided or discontinued (16). Contrast induced AKI, although uncommon in this age group, should be considered in neonates who undergo multiple radiographic studies.

**Table 5:** Common nephrotoxic medication used in neonates

**Medication**	**Toxicity**	**Monitoring Strategies**	**Uses**
Aminoglycosides	Nephro/ototoxic	Trough levels should be routinely monitored	Antibiotics
Ibuprofen	Nephrotoxic	Serum Creatinine and urine output should be normal before starting therapy	Used for treating PDA
Vancomycin	Nephrotoxic	Levels should be monitored	Antibiotics
Indomethacin	Nephrotoxic	Serum Creatinine and urine output should be normal before starting therapy	Used for treating PDA

## Hypotension and poor renal perfusion

There are multiple reasons for systemic hypotension leading to renal hypoperfusion in the NICU population (see Table 6) (54-57). Although a detailed description in the management of hypotension in newborns is beyond the scope of this review, the following are extremely important issues in management, as most of these conditions are unique to this population. Before the exact cause of hypotension is identified, it is important to ensure normal intravascular volume and start the child on vasopressors (54-56). Unrecognized and untreated hypotension can lead to renal hypoperfusion, ultimately resulting in AKI. Birth asphyxia is another common cause of AKI in newborn infants (16,26,31).

**Table 6:** Hypotension and renal hypoperfusion in NICU population

**Cause of hypotension**	**Mechanism/type of shock**	**Management**
1. Hemorrhage (placental abruption, cord avulsion, massive intraventricular hemorrhage (IVH), adrenal hemorrhage, hepatic subcapsular hematoma, retroperitoneal bleeding, surgical blood loss)	Hypovolemia	- Intravenous fluids - Packed red blood cell (RBC) transfusion
2. Sepsis	Distributive or Cardiogenic	- Antibiotics - Correction of fluid deficits - Vasopressors
3. Patent ductus arteriosus	Diastolic run-off (low diastolic blood pressure)	- Medical/surgical closure
4. Adrenocortical insufficiency	- Low Cortisol leading to vasopressor resistant hypotension	- Hydrocortisone
5. Necrotizing enterocolitis	Systemic inflammatory response leading to distributive shock	- Intravenous fluids - Vasopressors
6. Cardiogenic (congenital heart disease, myocarditis, pericardial effusion)	Cause specific	- Cause specific (inodilators, PGE2 if due to ductus dependent cardiac anomaly)

## Clinical consequences of AKI in neonates

### Electrolyte imbalances

Dilutional hyponatremia, hyperkalemia, hyperphosphatemia, hypocalcemia, hypomagnesemia, acidosis and uremia are fairly common, especially in the initial acute and often oliguric phase of illness (47). Moreover, hypokalemia and alkalosis can become an issue later on.

### Poor nutrition

During the acute phase of AKI, nutrition is often sub-optimal due to fluid restriction, and the catabolic phase may contribute to the increased blood urea nitrogen (BUN) (10).

### Fluid overload

Independently associated with increased mortality, fluid management becomes extremely challenging in sick, preterm infants with AKI (21). The sheer volume of medications can easily exceed the basal fluid requirement. Determining fluid balance is based on careful documentation of weight gain or loss, urinary output, serum sodium, BUN and urinary composition (sodium, osmolarity and FENA) (16). The goal is to maintain euvolemia and homeostasis until the renal functions recover, while maintaining adequate nutrition and oxygen delivery to the peripheral tissues. Multi-organ failure is the leading cause of death in neonates with renal failure (36,38). Additionally, fluid overload in an oliguric patient leads to pulmonary edema resulting in increased ventilator settings to offset the effect of the stiffening lungs (21,36). The resultant increase in the mean airway pressure leads to decreased venous return to the heart and exacerbates the poor renal perfusion (58).

Extravasation of the intravascular fluid (third-spacing) is common in fluid overloaded neonates (36) due to combination of fluid overload, low oncotic pressure and leaky capillaries secondary to sepsis and inflammation. The extravasated fluid not only depletes the intravascular fluid volume, but also causes skin breakdown and chest wall edema resulting in higher ventilatory requirements, and in extreme cases, fluid overload may lead to abdominal compartment syndrome (59). Skin integrity in extremely preterm infants is especially critical for prevention of insensible losses as it functions as an innate barrier for prevention of infections (36).

## Prevention

The causes of AKI in neonates are numerous. Additionally, a sick newborn is often exposed to multiple nephrotoxic agents. Identifying these risk factors and eliminating exposure to the avoidable ones is the first step in the management of AKI in newborns. Nephrotoxic medications commonly used in this population include aminoglycosides, vancomycin, ibuprofen or indomethacin (60), and amphotericin B (61). Efforts should be made to avoid these medications or, if they are absolutely indicated, then levels should be closely monitored (Table 5). Additionally, adequate management of conditions that result in poor renal perfusion such as systemic hypotension, hypovolemia, PDA, sepsis and hypoxemia are important in the prevention of established AKI (Table 6). Most cases of oliguria in neonates are associated with either decreased provision or increased loss of fluid (pre-renal). Common examples of decreased fluid provision include underestimation of fluid requirements and fluid restriction due to pulmonary edema or PDA. Also, certain congenital anomalies, such as gastroschisis and meningomyelocele, in which the viscera are not covered with skin, can lead to excessive insensible fluid losses. Finally, the use of diuretics is another common cause of fluid losses resulting in intravascular volume depletion.

## Non-dialytic management of AKI in the newborn

### Goals

The goal of AKI management in the newborn is to maintain homeostasis (fluid, electrolyte, nutrition and acidosis) until renal functions return. After the AKI diagnosis has been established, the identification of the cause is important to remove a reversible cause(s) of AKI. The causes of poor renal perfusion listed above should be identified and corrected (35,46). Next, renal ultrasound with Doppler should be used to look for congenital anomalies of the kidney and urinary tract (CAKUT), renal vascular conditions and bladder outlet obstruction should be performed in any newborn with oliguria/anuria (47). If an obstruction is identified, it should be promptly relieved by catheterization (urethral or suprapubic) and urological consultation should be sought for further management (47). Once an outlet obstruction is ruled out, careful assessment of the fluid balance should be made.

The following factors are taken into consideration in order to assess the status of the intravascular volume in cases of a sick neonate:

1. Weight gain or loss; the newborn should be weighed on a sensitive scale, preferable twice a day. The weight gain per day is quite variable in a newborn and depends on multiple factors like gestational age, post-natal age, degree of illness, and nutritional status (62). Excessive weight gain (more than 20-30 g/day) in a sick child is a sign of retained fluids; similarly, weight loss is a sensitive marker for negative fluid balance (21).
2. Vital Signs; tachycardia and low blood pressure (BP) are signs of hypovolemia (63). Other causes leading to increased heart rate (sepsis, fever, pain, medications, etc.) should be kept in mind, and low BP should be treated to preserve renal perfusion (35).
Serum and urinary chemistries should be obtained. Serum sodium can be a very sensitive marker for fluid status and is very important for management (47). The interpretation of serum sodium should be made carefully and take the following into consideration: sodium intake over the last few days, weight change, urinary output, serum BUN/Cr, urinary sodium and osmolarity. Hyponatremia can be seen due to dilutional effects of oliguric AKI or SIADH; other common causes include diuretic use and decreased amount of supplementation (35).
3. Combination of weight change, assessment of the past few days’ input and output log, change in the vital signs, and the serum and urinary chemistry is used to determine the fluid status (10). Since major causes of oliguria are pre-renal (see above), response to 20-40 mL/kg of crystalloid may help differentiate between pre-renal azotemia and an established oliguric AKI (35). The urine output should be critically measured by placing an indwelling urinary catheter (preferable) or urinary collection bag. If those are not possible, careful weighing of wet diapers every 3 hours is a less accurate, but acceptable, method of documenting the urinary output in mL/kg/h (26).
4. Alternative methods used to validate the intravascular fluid status include cardiac echo looking at the ventricular filling. The use of central venous pressure monitoring is difficult especially in very small neonates (54,64).

Based on the above, if the child is considered to be hypovolemic fluid challenge should be attempted.

## Estimation of fluid requirement

The goal of management in an oliguric/anuric patient is maintenance of the fluid and electrolyte balance and provision of adequate nutrition until renal functions recover. While managing the fluid status of a newborn, the following should be carefully considered:

1. During the first week of life, term newborns loose about 5%-10% body weight due to the shrinkage of the extracellular fluid compartment (65). The physiological weight loss may be up to 14% in preterm infants (65).
2. The daily fluid requirement is based on estimation of insensible water losses plus ongoing losses (e.g. surgical drains) and the previous day’s urinary output. The fluid requirement in term infants is dependent on the day of life and presence of any congenital anomalies that would result in excessive fluid loss. In general, the insensible loss of fluid from skin and respiratory tract is 40-50 mL/kg/day (10,66).
3. In preterm infants, insensible losses are estimated based on the skin maturity (keratinization), day of life, use of ambient humidity in the isolette, and ongoing losses (drains, gastric losses and urinary output) (65,66). Based on the weights, newborns < 750 g would have 100-150 mL/kg/day of insensible losses while newborns weighing 750 g to 1000 g would receive 60-70 mL/kg/day. More mature preterm newborn weighing between 1000-1250 g would have 30-65 mL/kg/day of insensible losses (65,66).
4. Fluid overload may not only cause third spacing, pulmonary edema, increased respiratory requirements and worsening of the PDA, but may also result in dilutional hyponatremia due to excessive free water (21,35). Because of massive fluids shifts, contraction of the extracellular fluids and natriuresis that accompanies the physiological weight loss during the first few days, sodium is not added to TPN for the first 48 to 72 hours (65). Thereafter, 2-3 mEq/kg/day can be added slowly to the fluids. It is important to account for the inadvertent administration of sodium along with umbilical arterial line fluid and other medications (35,65,66).

## Management of electrolyte imbalances

**Sodium:** Serum sodium should be monitored closely, based on basal requirements of 2-4 mEq/kg/day of sodium, and the daily provision adjusted based on estimated ongoing losses. Dilutional hyponatremia is common in AKI and should be corrected by restricting the free water provision (47). In addition, sodium concentrations should be normalized prudently in order to prevent negative neurological outcomes (47).

**Potassium:** Hyperkalemia is common in the oliguric and anuric phase. Therefore, all potassium-containing fluids should be eliminated, or discontinued as soon as oliguria or hyperkalemia is encountered. In the recovering polyuric phase, hypokalemia can become an issue and must often be corrected (35,67). Commonly used strategies for treating hyperkalemia have to be modified in preterm infants.

Hyperkalemia should be confirmed by venous sample, as hell-stick samples are often erroneous due to hemolysis (67). Preterm infants tolerate hyperkalemia well, and therefore electrocardiogram (EKG) changes may not be reliable and may not be seen for high potassium levels (67). Kayexalate has been used as an exchange resin in neonates and can be administered rectally as an enema. There have been case reports documenting intestinal complications both with oral and rectal use (68,69). There have been concerns about its use as an enema since sorbitol, which is used for the suspension, results in high osmolarity leading to NEC and colonic perforation (70). Although the water suspension of Kayexalate is thought to be safe for enemas in preterm infants, there has been a reported case of colonic perforation (69). Salbutamol can be used in acute management of dangerous hyperkalemia and may be safer than kayexalate in preterm infants (68). The usual strategies of calcium gluconate infusion, sodium bicarbonate infusion and insulin/dextrose infusion have to be tailored according to the patient’s clinical condition. Bolus administration of sodium bicarbonate has been associated with sudden changes in the osmolarity and pH, leading to an increased incidence of intra-ventricular hemorrhage (IVH) (71). Therefore, it should be used as a slow infusion and with great caution in preterm infants at risk for IVH (71). Insulin should be used very carefully and only as an infusion since hypoglycemia may lead to life-long neurodevelopmental impairments.

**Calcium/Phosphate:** Hyperphosphatemia is commonly seen in the acute phases due to renal insufficiency in addition to hypocalcemia (10). Phosphate intake should be curtailed during the anuric phase followed by careful monitoring and slow reintroduction after the establishment of urinary output. Secondary hyperparathyroidism is rarely seen in the neonatal population.

**Renal trace elements:** Traditionally, kidneys predominantly excrete trace elements, like selenium and iodine. Chromium may have adverse effects on renal functions and is eliminated from the total parenteral nutrition (TPN) in patients with renal insufficiency (72).

## Nutrition

### Protein intake

Conventionally, protein intake is restricted in patients with increasing BUN because restriction has been proven to decrease the BUN in adult patients (73). During the past decade, however, neonatal nutrition has undergone a paradigm shift with respect to protein intake, and it is commonly thought that the growth failure in preterm newborns can be prevented by the provision of 3-4.5 g/kg/day of protein (74-76). It has now become routine practice to administer 3-4 g/kg/day of protein beginning within a few hours of birth (74,77). Poor nutrition not only affects the somatic growth but also leads to undesirable neurodevelopmental outcomes. Protein intake should be adjusted at least to meet the basal growth requirements (1-2 g/kg/day) while keeping the BUN below the threshold for causing increase in the serum osmolarity.

### Glucose

Hyperglycemia should be avoided and may have to be treated with insulin. Due to the lack of hepatic reserves and gluconeogenesis, preterm infants require an infusion of glucose 4-6 mg/kg/min to meet the obligatory glucose requirement of the brain (74). With fluid restrictions in place, infusion of a high concentration of glucose (> 12.5% Dextrose) can only be achieved via a central catheter. Parenteral glucose and intra-lipids are the predominant source of calorie intake in preterm infants. Additionally, in the absence of adequate calorie intake, dietary proteins are oxidized for energy instead of being used for tissue synthesis. This oxidation of proteins further contributes to the rising BUN. Hence, adequate caloric provision is not only important for anabolism but also for the rise of BUN (73). The daily caloric needs of infants should be calculated based on gestation and postnatal age. TPN should be tailored to meet both the fluid and caloric need (74,78).

Per the European Society of Pediatric Gastroenterology, Hepatology and Nutrition Guidelines (ESPGHAN) (79), the basal energy requirements of a newborn are estimated to 50-60 kcal/kg/day. For optimal growth and protein accretion, a growing preterm infant would require 100-120 kcal/kg/day. The caloric requirements for infants who are on TPN are estimated to receive 90-100 kcal/kg/day due to a lack of fecal losses and diet induced thermogenesis (74). The caloric needs are met through the parenteral route since most of these infants are clinically unstable. Delivery of adequate calories in a fluid restricted neonate may require placement of a central line since the maximum concentration of dextrose that can be infused through a peripheral catheter is 12.5%.

## Management of hypotension

There is a lack of broad consensus about the management of BP in premature newborns (54). This is partly due to the scarcity of normative data and partly due to the lack of evidence of overall benefit in the treatment of borderline low BP in absence of signs of tissue hypoperfusion (56,80). The normal values of BP vary with gestational and postnatal age (hours after birth) (55,57), but the most commonly used (although not always accurate) method for determining normal mean arterial pressure (MAP) is the gestational age in weeks.

The cause of hypotension in the neonate would determine the treatment. Hypovolemia should be corrected carefully since rapid boluses have been associated with increased cerebral blood flow and may result in IVH (71). Dopamine has been traditionally used as the first line vasopressor, followed by dobutamine in non-responsive cases. Norepinephrine and epinephrine infusions are useful in cases of peripheral vasodilatory states like sepsis. Hydrocortisone has been shown to be effective in cases of vasopressor resistant hypotension. Random cortisol levels are of questionable significance in determining the cortisol response in a sick neonate. Cardiogenic shock has to be managed according to the etiology. Diastolic run off through the PDA leading to hypotension would be an indication for medical or surgical treatment of the PDA. The data is much more conclusive in cases of full term infants.

BP can be monitored invasively by transducing an arterial catheter or non-invasively by using an automatic oscillatory BP instrument. Near infrared spectroscopy (NIRS) has been validated as accurate in determining the adequacy of cerebral and renal perfusion (81,82). Though not commonly used at present, it may become a non-invasive method of documenting tissue level hypoperfusion in the future (64).

## Role of diuretics

As mentioned earlier, fluid overload has proven to be an independent risk factor associated with higher mortality in late preterm neonates (21). It is also one of the most common indications for RRT in addition to electrolyte imbalance and metabolic disorders. Osmotic diuretics should be avoided due to unintended consequences such as risks for IVH, although loop diuretics are commonly used in preterm infants to treat bronchopulmonary dysplasia (BPD) and have a reasonably safe adverse effect profile (47).

Loop diuretics are used in the adult and pediatric population to treat fluid overload and convert oliguric renal failure to non-oliguric renal failure (47). Although the impact of loop diuretics on the outcomes of oliguric renal failure is debatable, urinary output allows the avoidance of fluid overload (83-85). Loop diuretics have also been proclaimed to be renal protective due to their effects on the redistribution of the renal blood flow resulting from the changes in the prostaglandin synthesis.

Loop diuretics should be used with caution due to their ototoxic potential (although reversible) (86,87) and the risk of renal calculi with long-term usage. Due to brisk diuresis, loop diuretics may be associated with sudden decrease in the intravascular volume and electrolyte imbalances like hyponatremia, hypokalemia and hypocalcemia (88). There are only a handful of cases reporting the use of diuretics in neonates with oliguric AKI (89-91), and long-term outcomes have not been reported. Currently, the evidence suggests that loop diuretics should not be used to prevent AKI, although in cases of fluid overload with oliguria/anuria they do provide a reasonable therapeutic option in the absence of RRT (47,92).

## Role of dopamine

Dopamine is a commonly used vasopressor in the neonatal population and has been shown to be safe and efficacious as a first line medication for treating hypotension in preterm infants (93). Dopamine is a catecholamine and has dose dependent effects on the systemic and renal vasculature (47). At lower doses it has been shown to improve renal perfusion through the stimulation of D1, D2, and D4 receptors (35). Though dopamine has not been shown to have a substantial impact on the outcome in adult and pediatric populations, it has been shown to transiently improve urinary output and serum creatinine in neonates (94). Only a handful of studies have documented improvement in urinary output in healthy preterm infants (95). Furthermore, despite the lack of evidence for substantial clinical benefits, low dose dopamine is anecdotally used in newborns with low urinary output (96).

## Role of dialysis

Use of RRT to treat AKI in the newborn, especially very low birth weight infants (<1500 g BW) is technically challenging and is not routinely attempted. There are occasional reports describing RRT use in such newborn infants (8,12). The indications for RRT are not absolute and are listed in Table 7. However, the detailed description is beyond the scope of this review.

**Table 7:** Indications for renal replacement therapy

**Indication**	**Peritoneal Dialysis efficacy**	**Hemodialysis efficacy**
Fluid Overload: Resulting in increased ventilatory support, nutritional compromise due to fluid restriction.	Good	Excellent
Hyperkalemia non responsive to medical management	Fair	Excellent
Hyperammonemia	Fair	Excellent
High blood urea nitrogen (BUN) and creatinine	Fair	Excellent
Congenital anomalies resulting in end stage renal disease - including CAKUT, poly/multi -cystic kidneys, inborn errors of metabolism, oxalosis, angiotensin receptor blockade fetopathy	Can be used for short term use	Long term

## Duration of renal dysfunction

Although prognosis and overall mortality is determined by the associated co-morbidities, infants with oliguric ARF have almost twice (81%-89%) the mortality rate of infants with non-oliguric renal failure (1,34,36,38,60). The following risk factors are associated with poor prognosis in cases of AKI in newborns: oliguria and anuria, multi-organ involvement, low gestational age, and low birth weight (9,12,21,38). Mortality has been reported to be low in cases of non-oliguric infants, and in few cases of neonates that have been treated with RRT. However, high mortality rates have been reported in studies examining neonates with established AKI (36,38). The outcome data is not much better with use of RRT (9,12,38). Duration of renal dysfunction and long-term outcome depends on the underlying cause. Children born with congenital anomalies may have permanent loss of function, while those who sustain AKI due to non-congenital causes tend to recover. The reported duration of renal dysfunction/oliguria is quite variable. On average, a newborn without congenital anomalies who sustained an acute insult resulting in AKI and oliguria would recover in 8 to 10 days, provided the inciting agent was removed and there were no ongoing nephrotoxic insults. Recent case series report that such newborns continue to be at high risk for renal dysfunction 3 to 5 years following the AKI episode (97). Another study reported up to 10% incidence of CKD among the survivors of AKI in the pediatric population (98). In the absence of robust follow-up data, it seems prudent to closely monitor the children who have survived AKI as neonates for development of CKD.

## Conclusion

Newborn patients with AKI present unique challenges when determining appropriate, patient specific treatment strategies. Although the goals (i.e. management of electrolyte imbalance) are similar when treating AKI in adult and pediatric patients, the steps taken to achieve such goals differ between these patient populations. Technical limitations imposed by small size of neonates especially very low weight infants often make RRT a nonviable mode of therapy for treating AKI in the newborn. Therefore, non-dialytic management of AKI not only addresses specific kidney needs, but also BP and nutritional needs required for normal growth and development of newborn patients.

## Authors’ contribution

All authors have contributed in the preparation of this paper. All authors have contributed in revising the paper and have read the revised version of the manuscript.

## Ethical considerations

Ethical issues (including plagiarism, data fabrication, double publication) have been completely observed by the authors.

## Conflicts of interest

The authors declare no conflict of interest.

## Funding/Support

None.
